# Hepatitis C Epidemiology: Insights from a Comprehensive Cohort Study in ASST Melegnano and Martesana, Lombardia Region, Northern Italy

**DOI:** 10.3390/pathogens13030215

**Published:** 2024-02-28

**Authors:** Michele Nardone, Dario Di Stasio, Alberta Lucchese, Daniele Gentili, Giulia Cattabianchi, Carlo Signorelli, Pierangelo Sarchi, Giovanna Pulcrano, Valentino Lembo, Paola Maria Pirola, Dorina Lauritano, Francesco Carinci

**Affiliations:** 1 Azienda Socio-Sanitaria Territoriale Melegnano Martesana, Regione Lombardia, 20077 Milan, Italy; michele.nardone@asst-melegnano-martesana.it (M.N.); pierangelo.sarchi@asst-melegnano-martesana.it (P.S.); giovanna.pulcrano@asst-melegnano-martesana.it (G.P.); valentino.lembo@asst-melegnano-martesana.it (V.L.); paola.pirola@asst-melgnano-martesana.it (P.M.P.); 2 Multidisciplinary Department of Medical-Surgical and Dental Specialties, University of Campania “Luigi Vanvitelli”, 80138 Naples, Italy; dario.distasio@unicampania.it (D.D.S.); alberta.lucchese@unicampania.it (A.L.); 3 IRCCS San Raffaele Hospital, Vita-Salute University, 20132 Milan, Italy; gentili.daniele@hsr.it (D.G.); cattabianchi.giulia@hsr.it (G.C.); signorelli.carlo@hsr.it (C.S.); 4 Department of Translational Medicine, University of Ferrara, 44121 Ferrara, Italy; dorina.lauritano@unife.it

**Keywords:** HCV, screening, virus

## Abstract

Hepatitis C virus (HCV) infection is a significant public health problem affecting 58 million people worldwide, including 3.9 million in Europe. Many of these infections go undiagnosed because chronic infection is often asymptomatic. This observational cohort study presents a detailed examination of hepatitis C virus epidemiology in Lombardia (Italy) and was conducted within the ASST “Melegnano e della Martesana”. The study involved comprehensive HCV screening of 3290 patients accessing the collection points and/or hospitalized in the facilities of the ASST from 20 May 2022 to 13 April 2023. Screening was conducted using serological assays. The prevalence of anti-HCV-positive patients (HCV-Ab) and then HCV-positive patients (RNA) was calculated. Chi-square tests examined the associations between continuous and categorical variables. Logistic regression was used to evaluate the influence of demographic and geographic variables as predictors of HCV positivity. The study revealed an overall HCV-Ab prevalence of 0.912% (CI (0.59–1.24%)) in the examined cohort, of whom 15.15% (two females and three males) were positive for HCV RNA. The prevalence of HCV RNA positivity was 0.152% (CI (0.05–0.35%)). Sex disparity was evident, with male patients exhibiting a higher prevalence compared to females, confirmed by logistic regression (0.0147 vs. 0.0061–OR = 2.44; CI (0.0059–0.0124)). Age stratification indicated an ascending trend in prevalence with age, peaking at 1.35% in individuals aged over 50. These findings underscore the critical need for targeted HCV screening, contributing valuable insights to the global epidemiology of HCV in the era of DAAs.

## 1. Introduction

Viral hepatitis caused by the hepatitis C virus (HCV) is a significant public health problem affecting 58 million people worldwide, including 3.9 million in Europe. Many of these infections go undiagnosed because the chronic infection is often asymptomatic [1,2,3,4].

HCV infection causes an acute, often mild or asymptomatic disease that tends to persist in the body, becoming chronic in 50–80% of those infected [5]. The evolution of the disease in 20% of cases can lead to the development of cirrhosis, hepatocellular carcinoma, and consequently death [6].

HCV is a blood-borne virus. In the world, the most common risk factors associated with the infection are the use of injection drugs (among people who inject drugs (PWID)), exposure to unsafe diagnostic or therapeutic procedures, and transfusions of unscreened blood and blood products leading to blood exposure. Sexual transmission is also a recognized risk factor. Vertical transmission from infected mother to child is also possible [1,2,3,7]. In 2019, hepatitis C resulted in an estimated 12,696 deaths. In Italy, an estimated 1554 died in 2019 [8].

### 1.1. Hepatic and Extra-Hepatic Manifestations in Hepatitis C Virus Infection

Hepatitis C virus (HCV) infection has a wide array of clinical manifestations extending beyond the liver (hepatic) to involve various other organs and systems (extra-hepatic) [9]. This multidimensional impact of HCV significantly complicates the clinical course and management of the infection.

Chronic HCV infection is primarily known for its hepatic consequences, which range from mild, asymptomatic chronic hepatitis to more severe forms like cirrhosis and hepatocellular carcinoma (HCC). The pathogenesis of liver injury in HCV is complex, involving direct viral cytopathic effects, immune-mediated mechanisms, and metabolic disturbances. Chronic inflammation triggered by the virus leads to fibrosis, which gradually progresses to cirrhosis. Patients with cirrhosis are at a higher risk for developing HCC, a leading cause of cancer-related deaths globally. The risk of HCC persists even after achieving sustained virological response (SVR), necessitating ongoing surveillance in patients with advanced fibrosis or cirrhosis [10].

HCV is also associated with a range of extra-hepatic manifestations that affect various organ systems. The understanding of these extra-hepatic effects is crucial, as they often precede hepatic symptoms and can be the initial clue leading to HCV diagnosis. These manifestations are primarily immunologic in nature, arising from chronic immune activation, and include mixed cryoglobulinemia, lymphoma, thyroiditis, Sjögren’s syndrome, and diabetes mellitus [11,12,13,14,15].

HCV can also affect the central nervous system, leading to a range of neurological and psychiatric disorders [16], and it is associated with a variety of rheumatologic conditions [17] and skin conditions [18,19,20]. In summary, the extra-hepatic manifestations of HCV are varied and can involve multiple organ systems. These manifestations significantly influence the clinical approach to HCV, emphasizing the need for a multidisciplinary care approach. Adequate screening and management strategies for these conditions are essential in the overall care of patients with HCV, alongside the focus on liver disease.

HCV hepatitis represents the only chronic viral infection that can be cured with antiviral drugs. Thanks to the introduction of new therapies based on pangenotypic direct-acting antivirals (DAAs), it has been possible to achieve eradication of the infection in more than 90% of affected individuals. The necessary strength of these treatments is the low occurrence of side effects [21,22].

Treatment for HCV has brought gains in Italy in terms of reductions in infection-related clinical complications, such as liver cancer, liver failure, and the need for transplantation [22,23,24].

Despite the lack of a vaccine, the extraordinary efficacy of these drugs prompted the World Health Organization to formulate an action plan to include the elimination of HCV infection as a global health goal for 2030, defining a 65 percent decrease in mortality and a 90 percent reduction in new infections as targets to be achieved [25].

### 1.2. Screening Legislation

Decree-Law 162 of 30 December 2019, implemented by the “Mille proroghe Decree” of 28 December 2020 and the State-Regions Conference of 17 December 2020, regulated screening interventions for active HCV infection in order to enhance the campaign for the global elimination of this infection by 2030. The screening project was effectively implemented in the Ministry of Health Decree of 14 May 2021 [26,27,28].

According to this decree, the screening:

- Is carried out with the intent to detect as yet undiagnosed HCV infections, improve the possibility of early diagnosis, initiate patients to treatment, as well as interrupt the circulation of the virus by preventing new infections;
- Shall be carried out by serological testing, by detection of anti-HCV antibodies (HCV-Ab) and/or reflex testing;
- Shall be carried out after appropriate information is provided to the individuals concerned by healthcare providers. Written informed consent to the performance of the test and processing of personal data must be acquired [28].

The Directorate General (DG) Welfare of the Lombardy region implemented this decree with Resolution XI/5830, “Program for the implementation of the plan for the elimination of HCV in the Lombardy region.” This program stipulates that the tests are to be carried out in conjunction with a blood draw as part of hospital admissions (ordinary and day hospital) and at the time of blood test withdrawals. Screening is to be carried out by any contracted accredited public or private healthcare facility. Each facility reports to a hepatitis C specialist center.

The screening campaign is aimed at all individuals enrolled in the health registry, resident, domiciled, or assisted in the Lombardy region.

Being in treatment or having already been treated with DAAs or having already participated in a screening are criteria for exclusion from screening.

All Aziende Socio-Sanitarie Territorialis (ASSTs), hospitals, and blood collection points in the Lombardy region participate in screening and are all considered first-level screening points, that is, they perform venous blood sampling for HCV antibodies.

If the first-level test is positive, the second-level test for HCV-RNA is performed. Testing for HCV-RNA is performed on a second venous collection or on the blood sample used for the first-level test by reflex testing.

The first-level center is responsible for offering the test according to the modalities defined for the specific setting (outpatient or inpatient) and for verifying the criteria for exclusion from screening. Each screening point is responsible for the maintenance and reporting of data and their proper uploading to the platform prepared for this purpose by the DG Welfare of the Lombardy region.

For the implementation of screening, the DG Welfare of the Lombardy region has produced an informed consent form and a consent form for the processing of personal data that must be provided to users and signed by them in order for them to be able to join.

### 1.3. Objectives

The main objective of this cohort study was to calculate the prevalence of HCV infection through screening in a population sample that has access to specific ASSTs in the Lombardy region of Italy.

## 2. Materials and Methods

### 2.1. Study Design and Patient Recruitment

The screening was conducted in the facilities of the ASST “Melegnano e della Martesana”, which provides healthcare in 53 cities in the southeastern province of Milan, with about 650,000 residents. The ASST consists of a hospital hub and a territorial hub, composed of 5 hospital facilities, 14 multi-specialty facilities, and 23 district social-health facilities. Collection points are located at the 5 hospital establishments and 6 multi-specialty facilities. Screening is offered to users accessing the collection points (outpatient setting) and hospitalized (hospital setting) in the facilities of the ASST Melegnano e della Martesana.

The screening test is performed by testing blood for HCV antibodies (HCV–Ab; first-level test). In case of a positive result, HCV-RNA is tested for diagnostic confirmation (HCV RNA; second-level test) using the same blood sample (reflex testing) in the ASST testing laboratory.

The screening campaign is aimed at all individuals enrolled in the health registry, resident, domiciled, or assisted in the Lombardy region, born between 1969 and 1989 (inclusion criteria).

Exclusion criteria for the screening are being in treatment or having already been treated with DAAs or having already participated in a screening.

A cohort study was conducted, following the STROBE guidelines to ensure comprehensive reporting of observational epidemiology. Patients were recruited consecutively from 20 May 2022 to 13 April 2023. Patients were categorized into the following age groups: 30–39, 40–49, and ≥50. Eleven geographic areas were identified, each comprising different facilities: Cassano withdrawal point (Cassano); Cernusco ward, Cernusco withdrawal point (Cernusco); Gorgonzola withdrawal point (Gorgonzola); Melegnano withdrawal point (Melegnano); Melzo ward, Melzo withdrawal point (Melzo); Paullo withdrawal point (Paullo); Peschiera withdrawal point (Peschiera); San Donato withdrawal point (San Donato); San Giuliano withdrawal point (San Giuliano); Vaprio withdrawal point (Vaprio); Vizzolo ward, Vizzolo pre-hospitalization, Vizzolo withdrawal point (Vizzolo).

The demographic and geographic variables collected are reported in Table 1.

### 2.2. Screening and Diagnostic Confirmation

Screening for hepatitis C antibodies (anti-HCV) was performed using serological assays, such as Enzyme Immunoassays (EIAs), Chemiluminescent Immunoassays (CLIAs), and Rapid Immuno-Chromatographic Tests (ICTs). Positive anti-HCV results were subsequently confirmed with HCV RNA testing to verify active viral infections. The analyses were performed in the ASST laboratory.

### 2.3. Statistical Analysis

Statistical analysis was conducted using RStudio (v. 2023.09.1 + 494).

The prevalence of HCV was computed by dividing the number of positive test outcomes by the total number of individuals tested, expressed as a percentage. This analysis was stratified based on age groups and sex to understand the distribution patterns of HCV infection.

The prevalence of HCV infection was also calculated by geographic area. The Student’s *t*-test was used for determining the differences between the age groups by sex; Pearson’s correlation and chi-square tests examined the associations between continuous and categorical variables. Logistic regression models were utilized to evaluate the influence of demographic and geographic predictors on HCV positivity.

## 3. Results

The sample analyzed in this study consisted of 3290 patients, of whom 2135 were females (64.89%; mean age: 44.13 ± 5.99 years; range: 32–54; median: 45 years) and 1155 were males (35.10%; mean age: 44.87 ± 6.03 years; range: 32–54; median: 46 years), with males tending to be older than females (*p* < 0.001). The mean age of the total sample was 44.4 ± 6.01 years (median: 45; range: 32–54). All patients were screened for HCV antibodies. A total of 30 patients (13 females and 17 males) were found to be positive at the test, 5 (15.15%, two females and three males) of whose diagnoses were confirmed by reflex testing (HCV RNA).

The total prevalence of anti-HCV-positive cases was 0.912% (CI (0.59–1.24%)), while the prevalence of HCV RNA positivity was 0.152% (CI (0.05–0.35%)). Male patients revealed a higher prevalence of HCV antibodies (1.47%) than female patients, 0.61% (*p* = 0.022). The log-odds of being HCV-positive for males was 0.891 higher than for females (OR = 2.44; CI (0.0059–0.0124)).

The geographical area with the highest recorded prevalence was Peschiera, with 2 cases out of 72 patients screened in the various facilities and a prevalence of 2.53%, and Vizzolo (15/1033, 1.45%), followed by Paullo (2/150, 1.33%), Melzo (3/41, 70.72%), Vaprio (3/444, 0.68%), Gorgonzola (2/311, 0.64%), and Cernusco (3/635, 0.47%). All areas’ data are shown in Table 2.

**Figure 1:**
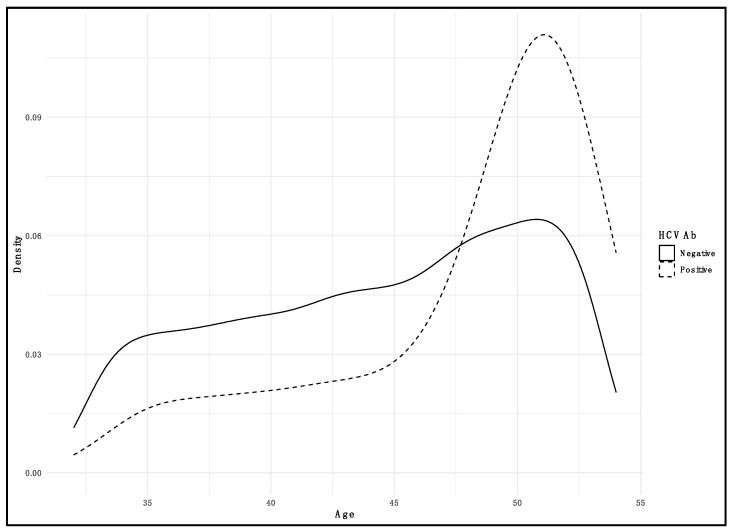
Density plot illustrating the distribution of HCV positivity among screened subjects by age. Solid line represents negative subjects; dashed line represents positive subjects.

The prevalence of HCV infection across different age groups was examined within the study cohort. In the youngest demographic, individuals aged under 40 (30–39), there were 833 total cases examined, among which four were positive for HCVAb, yielding a prevalence rate of 0.48%. For the 40–49 age group, out of 1603 total cases, there were 8 positive cases, resulting in a prevalence rate of 0.49%. The ≥50 age group showed the highest prevalence rate: out of 854 total cases, there were 18 positive cases, indicating a prevalence rate of 2.11% (Figure 1).

The logistic regression analysis revealed a significant association between age and HCV positivity (*p* < 0.05); with each additional year of age, the odds of being HCV Ab-positive increased by 12.3% (CI 95% (4.7–21.7%)).

Moreover, in the under-40 age categories, female subjects accounted for 559 total cases, with 1 HCV Ab-positive case (and no subject positive for HCV RNA), resulting in a prevalence of 0.18%, while males in the same age group showed 3 positive cases out of 274 total cases, with a prevalence of 1.10%. Among individuals aged 40–49, females had a total of 1058 cases, with 5 positive cases (and 1 HCV RNA-positive subject), leading to a prevalence of 0.47%; males had 545 total cases, with 3 positive cases (0.55%). In the 50–59 age group, females presented 518 total cases, with 7 patients positive for antibodies (1 confirmed), translating into a prevalence of 1.35%; the prevalence was higher among males in this group, with 11 positive cases (3 confirmed) out of 336 (3.27%) (Table 3).

## 4. Discussion

Our study presents an intricate portrait of HCV epidemiology across various demographics and geographic distributions within the Lombardia region, contributing to a global understanding of the disease’s prevalence. With a comprehensive cohort of 3290 individuals, our findings underscore the nuanced interplay between age, sex, and geography in HCV transmission dynamics.

The present study’s findings highlight the heterogeneity of HCV prevalence within Lombardia, both geographically and demographically. Our results indicate a total anti-HCV positivity rate of 0.912%, with notable variability across different areas and age groups. This prevalence is comparatively lower than the overall prevalence of 2.3% reported in the study by Andriulli et al. [29], which underscores the potential effectiveness of public health measures and treatment regimens currently in place in Lombardia.

The geographical disparities within Lombardia—with Peschiera exhibiting the highest prevalence of HCV Ab positivity at 2.532% and areas like Cassano, Melegnano, San Donato, and San Giuliano showing no cases—may reflect local differences in risk factor exposure or access to healthcare services. This variation emphasizes the need for region-specific strategies, a sentiment echoed by Kondili et al. [30], who also observed non-significant geographical differences in HCV prevalence across different Italian regions.

Age-wise, the increasing prevalence of HCV with advancing age observed in our cohort is consistent with the findings of Andriulli et al. [29], who demonstrated a peak in prevalence among the elderly. In our study, the highest prevalence was in the ≥50 age group, with a rate of 3.448% in males and 1.35% in females. This age-related trend stresses the importance of targeted screening programs for older populations, who may have been exposed to risk factors such as unscreened blood transfusions or unsterile medical equipment in the past.

Sex differences in prevalence were also apparent in our study, with males exhibiting a higher prevalence (1.47%) compared to females (0.61%). These findings are in line with the sex ratio (M/F) of 1.5 and higher prevalence in men found by other authors [29,31], suggesting a potential need for increased vigilance in male populations, especially considering occupational and behavioral risk factors that may contribute to these differences. The different prevalences found in the various areas were analyzed by the authors. Unfortunately, based on the data we have (age and gender), no correlations have been found to justify these differences.

Furthermore, the confirmation of active HCV infection through HCV RNA positivity in our study was lower (0.387%) than that based on anti-HCV positivity, and it was inferior to those indicated in the literature [29,30].

Our findings, while providing a snapshot of the HCV landscape in Lombardia, also contribute to the broader understanding of HCV epidemiology. The lower overall prevalence in our study compared to historical data suggests progress in combating HCV, likely due to improved screening and treatment efforts. Nonetheless, the persistence of higher prevalence in specific demographics and regions indicates ongoing challenges and the need for continued, and perhaps intensified, public health interventions.

In conclusion, the heterogeneity in HCV prevalence across age groups, sexes, and geographical regions demonstrated in our study emphasizes the need for tailored approaches to HCV screening and treatment. By integrating our data with the wider epidemiological context provided by the existing literature, we can refine our strategies to better target the populations most at risk and move closer to the goal of eradicating HCV.

The current study’s findings regarding the demographic patterns of HCV prevalence offer critical insights for public health strategies. The observed sex and age disparities in HCV prevalence underscore the need for targeted interventions. Specifically, the higher prevalence in males and older age groups suggests that screening programs should prioritize these populations. Additionally, the geographic variability in HCV prevalence within our study indicates the necessity for location-specific public health policies, which could include focused educational campaigns and tailored screening initiatives.

Furthermore, our findings have implications for the global efforts to eradicate HCV. The World Health Organization’s goal of eliminating HCV as a public health threat by 2030 requires a multifaceted approach, combining effective screening, treatment, and prevention strategies. The insights provided by our study contribute to this global endeavor, offering a nuanced understanding of the epidemiological landscape that can inform policy and resource allocation.

The silent progression of HCV Is ”arti’ularly insidious; patients often remain asymptomatic until the disease has advanced to a critical stage, which underscores the importance of widespread screening programs. Implementing such programs aims to identify and treat the broadest population segment possible, thereby reducing the disease’s prevalence and, potentially, leading to its eradication in the coming years. Several authors have indicated that HCV clearance performed with DAAs enhanced both liver disease and associated extrahepatic clinical manifestations [32,33,34,35].

Global epidemiological data illustrate a heterogeneous distribution of HCV, with prevalence rates varying significantly by geographic region. Certain areas, due to a combination of social, economic, and healthcare-related factors, exhibit higher endemicity [33]. For instance, in some regions of Asia and Africa, unsafe medical practices and limited access to healthcare infrastructure exacerbate the spread of HCV.

The advent of DAAs has revolutionized the treatment landscape of HCV, offering cure rates exceeding 95% [23,24]. However, the success of these treatments hinges on early detection through diligent screening. As we consider the future of HCV epidemiology, the strategic deployment of DAAs in concert with comprehensive screening programs could dramatically alter the trajectory of the disease.

This study contributes to the growing body of evidence supporting the strategic implementation of HCV screening programs, not forgetting that this model could be applied to many other viral infections [36].

Unfortunately, this study has several limitations. The first limitation is that it concerns a small cohort born between 1969 and 1989, limiting the comparison of epidemiological data with other studies that include larger population cohorts. Another limitation of the study is that only demographic data (sex and age) in relation to HCV infection were available for study. Unfortunately, this study involved several collection centers, and the database was obtained through a preset form that did not provide access to any other data. Moreover, no vulnerable or high-risk groups were included in this study, which might have led to the underestimation of the HCV burden in the region.

## 5. Conclusions

In summary, this study has provided valuable insights into the epidemiology and clinical manifestations of hepatitis C virus (HCV) infection, emphasizing the crucial role of comprehensive screening and management strategies.

Moreover, our research contributes to the global initiative to eliminate HCV as a public health threat. Achieving this goal requires not only effective treatment options but also robust public health strategies, including widespread screening, public awareness campaigns, and access to healthcare services. Continued research and policy efforts are vital to address the gaps in HCV screening, treatment accessibility, and patient education.

In conclusion, while significant strides have been made in the battle against HCV, our collective efforts in research, public health policy, and clinical practice must continue to evolve. Embracing a multifaceted approach is the key to mitigating the impact of HCV and marching towards its eventual eradication as a global health burden.

## Figures and Tables

**Table 1:** Demographic and geographic variables.

Area	Sex (Females/Males)	Total Screened	Age (Mean ± SD)
Cassano	31/20	51	42.77 ± 6.19
Cernusco	404/231	635	44.43 ± 6.15
Gorgonzola	205/106	311	44.31 ± 6.29
Melegnano	23/16	39	45.31 ± 6.04
Melzo	311/106	417	44.68 ± 5.62
Paullo	104/46	150	44.23 ± 6.33
Peschiera	53/26	79	44.70 ± 6.28
San Donato	63/36	99	43.47 ± 6.20
San Giuliano	23/9	32	42.31 ± 6.23
Vaprio	292/152	444	44.93 ± 5.87
Vizzolo	626/407	1033	44.23 ± 5.96

**Table 2:** Area-specific prevalences (%) calculated based on the number of positive results out of the total number of subjects tested. CI 95% (lower–upper).

Area	HCV Ab+	Prevalence	CI	HCV RNA+	Prevalence	CI
Cassano	0	0	–	-	-	–
Cernusco	2M/1F	0.472	0.0975–1.37	-	-	–
Gorgonzola	2F	0.643	0.0780–2.30	-	-	–
Melegnano	0	0	–	-	-	–
Melzo	3F	0.719	0.149–2.09	1F	0.24	0.0061–1.329
Paullo	2M	1.333	0.162–4.73	-	-	–
Peschiera	2M	2.532	0.308–8.85	-	-	–
San Donato	0	0	–	-	-	–
San Giuliano	0	0	–	-	-	–
Vaprio	1M/2F	0.676	0.140–1.96	-	-	–
Vizzolo	10M/5F	1.452	0.815–2.38	1F/3M	0.387	0.106–0.988

**Table 3:** Prevalence (%) by age group and sex. CI 95% (lower–upper).

Age Group	Sex	Screened	HCVAb+	Prevalence	CI 95%	HCV RNA+	Prevalence	CI 95%
30–39	F	559	1	0.179	0.005–0.993	–	–	–
	M	274	3	1.100	0.226–3.166	–	–	–
40–49	F	1058	5	0.473	0.154–1.10	1	0.095	0.0024–0.525
	M	545	3	0.550	0.114–1.600	–	–	–
≥50	F	518	7	1.350	0.545–2.764	1	0.193	0.0049–1.07
	M	336	12	3.448	1.645–5.782	3	0.893	0.185–2.59

## Data Availability

The data presented in this study are available on request from the corresponding author.
